# Comparison of outcomes and side effects for neoadjuvant chemotherapy with weekly cisplatin and paclitaxel followed by chemoradiation vs. chemoradiation alone in stage IIB–IVA cervical cancer: study protocol for a randomized controlled trial

**DOI:** 10.1186/s13063-021-05986-z

**Published:** 2022-01-10

**Authors:** Jing Li, Hua Liu, Ya Li, Jian Li, Lifei Shen, Wenqing Long, Chenmin Yang, Haoping Xu, Wenqi Xi, Rong Cai, Weiwei Feng

**Affiliations:** 1 Department of Gynecology and Obstetrics, Ruijin Hospital, Shanghai Jiaotong University School of Medicine, Shanghai, 200025 People’s Republic of China; 2 Department of Radiology, Ruijin Hospital, Shanghai Jiaotong University School of Medicine, Shanghai, 200025 People’s Republic of China; 3 Clinical Research Center, Ruijin Hospital, Shanghai Jiaotong University School of Medicine, Shanghai, 200025 People’s Republic of China; 4 Department of Radiotherapy, Ruijin Hospital, Shanghai Jiaotong University School of Medicine, Shanghai, 200025 People’s Republic of China; 5 Department of Oncology, Ruijin Hospital, Shanghai Jiaotong University School of Medicine, Shanghai, 200025 People’s Republic of China

**Keywords:** Dose-dense neoadjuvant chemotherapy, Weekly cisplatin/paclitaxel, Concurrent chemoradiotherapy (CCRT), Stage IIB–IVA cervical cancer

## Abstract

**Background:**

Currently, the standard treatment for locally advanced cervical cancer is concurrent chemoradiation (CCRT). The effect of neoadjuvant chemotherapy in advanced cervical cancer is controversial. Studies have shown that the addition of a weekly regimen of neoadjuvant chemotherapy (NACT) followed by CCRT may be superior to a thrice-weekly regimen of NACT and CCRT. Among patients who had not received prior cisplatin, a cisplatin and paclitaxel (TP) regimen resulted in longer overall survival than other regimens. This study aims to investigate the feasibility, safety, and efficacy of NACT with weekly TP followed by CCRT.

**Methods:**

This is a prospective, randomized, open-labeled, multicentered phase III study. Based on a 65% of 2-year disease-free survival (DFS) rate in the CCRT group and 80% of that in NACT followed by CCRT group, and on prerequisite conditions including an 8% loss to follow-up, a two-sided 5% of type I error probability, and an 80% of power, a total of 300 cases were required for enrollment. Patients with IIB–IVA cervical cancer will be randomly allocated in a 1:1 ratio to one of two intervention arms. In the study arm, patients will receive dose-dense cisplatin (40 mg/m^2^) and paclitaxel (60 mg/m^2^) weekly for 4 cycles followed by CCRT (45 Gy in 5 weeks concurrent with cisplatin 40 mg/m^2^ weekly) plus image-guided adaptive brachytherapy (IGBRT). In the control arm, patients will undergo CCRT treatment. The primary endpoint of the study is 2-year disease-free survival (DFS); the secondary endpoints are 5-year overall survival (OS) and disease-free survival (DFS), the response rate 3 months after treatment completion, grade III/IV adverse effects, and quality of life, and potential biomarkers for predicting treatment response will also be studied.

**Discussion:**

The data gathered from the study will be used to determine whether NACT with weekly TP followed by CCRT may become an optimized treatment for locally advanced cervical cancer.

**Trial registration:**

Chinese Clinical Trial Registry ChiCTR1900025327. Registered on 24 August 2019. medresman.org.cn ChiCTR1900025326

## Background

Cervical cancer, a considerable health crisis for women, is the fourth most common cancer worldwide and the fourth leading cause of cancer death [1]. The standard treatment for locally advanced cervical cancer (LACC) is currently concurrent chemoradiation (CCRT). However, the overall survival (OS) for stage IIB and III–IV cancer is approximately 60–65% and 25–50%, respectively, which are frustratingly low [2]. Therefore, it is imperative to develop new treatment strategies to improve survival.

Neoadjuvant chemotherapy (NACT) was first reported by Frei [3]. The aim of chemotherapy preceding local modalities is to reduce the volume of the disease, making subsequent irradiation or surgery more effective while controlling the micrometastatic disease. A comprehensive meta-analysis was performed including 4727 cases from 13 publications to precisely assess the prognostic role of NACT for LACC [4]. The response rate, specifically the clinical and pathological responses to NACT, ranged from 58.49 to 86.54% and 7.5 to 78.81%, respectively; the treatment response indicated that LACC was sensitive to chemotherapy. In addition, the combined analysis showed that a better clinical response and pathologic response to NACT were associated with favorable PFS and OS. Para-aortic (PA) spread in cervical cancer is associated with a poor prognosis, despite the use of optimal first-line treatment, including chemoradiation therapy with extended field radiotherapy (EFR). Studies [5, 6] have suggested that adjuvant chemotherapy leads to better outcomes than chemoradiation therapy alone especially in PA-spread cervical cancer patients, despite increased toxicity.

NACT plays a yet unproved role in cervical cancer treatment, particularly when followed by CCRT, where data are scarce [7]. Traditional thrice-weekly regimens of NACT followed by CCRT may not be superior to CCRT alone for the treatment of LACC [8–10]. Weekly regimens of NACT followed by CCRT may be superior to CCRT alone [8, 11, 12], and this approach is now being evaluated in a randomized trial in an international study (weekly paclitaxel and carboplatin for 6 weeks) (NCT01566240: INTERLACE trial) (http://www.clinicaltrials.gov). Among the patients who had not received prior cisplatin, TP resulted in longer overall survival than TC [13]. Therefore, we will conduct this RCT to verify whether NACT with weekly cisplatin and paclitaxel for 4 cycles followed by CCRT is superior to CCRT alone and as safe as CCRT.

## Methods

### Ethical approval

The study is being conducted in China. The study protocol was approved by Shanghai Jiaotong University School of Medicine affiliated with Ruijin Hospital Ethics Committee (N-2018-239). All the group members have GCP certificates. Any recorded results will be anonymized in our study database.

### Study design

This is a prospective, randomized, open-labeled, multicentered phase III study. This study aims to investigate the feasibility, safety, and efficacy of NACT with weekly TP followed by CCRT. The 2-year progression-free survival in stage IIB–IVA cancer patients treated with CCRT is approximately 65% according to the literature. The desired 2-year progression-free survival treated with NACT followed by CCRT will be 80%. We assume an 8% loss to follow-up, and we seek a power of 80% at a two-sided significance level of 0.05. To detect a 15% difference, we will need 150 patients (per group). Based on an institutional volume of 80 patients per year and 20 patients per year in other centers, this trial will require 3 years to complete the recruitment of 300 patients. The objective is to explore the overall survival rate, disease-free response rate, response rate 3 months after treatment completion, survival outcomes, quality of life, and severity of side effects of dose-dense NACT followed by CCRT. The potential biomarkers for predicting treatment response will be studied.

### Study organization

The study was designed by a team of researchers from Shanghai Jiaotong University Medical School affiliated with Ruijin Hospital. It is being conducted at 5 sites in China: Shanghai Jiaotong University Medical School affiliated with Ruijin Hospital, Renji Hospital, the First People’s Hospital, Shanghai Tongji University affiliated with Oriental Hospital, and Fujian Medical University affiliated with Union Hospital.

Our group and its individual members have substantive experience in conducting studies on LACC. There will be a coordinating principal investigator (CPI) in the coordinating center and a principal investigator (PI) for each center. The CPI will be responsible for overseeing the study, and the PIs will take care of the study at the center level. We have a multidisciplinary group to assess the stage and status of the patients precisely, to assure the patients meet the inclusion criteria, to treat the patients and monitor the side effects, and to follow the patients on a regular basis. Also, the endpoint adjudication committee will be in charge of assessing the clinical response of treatment, which comprises experienced radiologists and gynecological oncologists. An independent Data Safety Monitoring Board (DSMB) will periodically review the safety data for trials to ensure subject safety and manage the whole process. The data management team include statistician who will participate in the study design, data cleaning, and data analysis and CRCs with responsibilities of CRF recording and data quality monitoring.

### Participants

We plan to recruit adult patients who have cervical cancer with the following inclusion and exclusion criteria.

#### Inclusion criteria

Each participant must meet all the following criteria to participate in this study: (1) age, 18~70 years (71–75 years without chronic complications could also be enrolled); (2) histologically confirmed squamous carcinoma, adenocarcinoma, or adeno-squamous carcinoma of the cervix; (3) FIGO (2018) stages IIB–IVA as evaluated by two senior gynecological oncologists; (4) performance status, Eastern Cooperative Oncology Group (ECOG) ≤ 2; (5) body weight ≥ 40 kg; (6) adequate bone marrow function (WBC ≥ 4.0 × 10^9^/L, neutrophils ≥ 2.0 × 10^9^/L, HB ≥ 70 g/L, and platelets ≥ 100.0 × 10^9^/L), adequate liver function (serum bilirubin, ALT, AST, and ALP ≤ 1.0 N), and adequate renal function (defined as a GFR ≥ 60 mL/min calculated using the Wright equation and Cr ≤ 1.0 N); (7) no active tuberculosis; (8) no pregnancy or lactation; (9) negative human immunodeficiency virus (HIV) test; and (10) written informed consent before enrollment and before initiation of any procedure.

#### Exclusion criteria

Individuals who meet any of the following criteria will be excluded from the study: (1) previous pelvic malignant diseases treated with chemotherapy or radiotherapy, (2) pelvic or abdominal radiotherapy, (3) suprarenal lymph node metastasis or distant metastasis detected by PET/CT or chest/abdominal CT, (4) acute infection or uncontrolled severe medical, (5) pregnant or lactating, (6) intestinal perforation or acute ileus, (7) uncontrolled cardiac disease (defined as a cardiac function that would preclude hydration during cisplatin administration and any contraindication to paclitaxel), (8) adrenocortical insufficiency, (9) bone marrow metastasis, or (10) not meeting requirements for chemotherapy.

### Study procedures and treatments

**Fig. 1:**
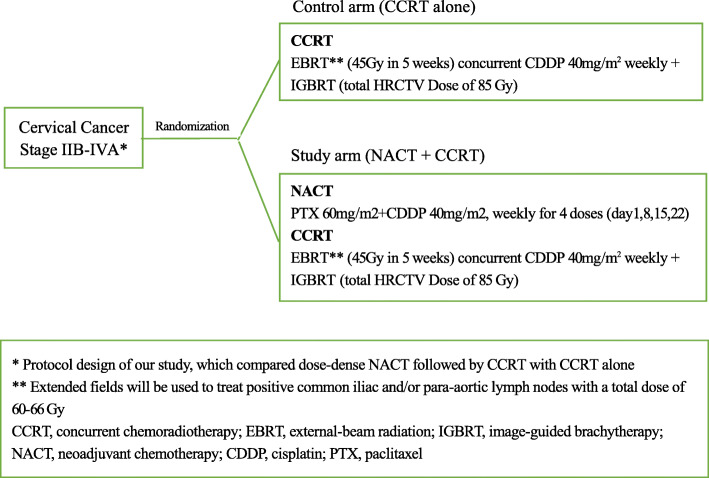
Protocol design (study procedures and treatments, paragraph 1)

**Fig. 2:**
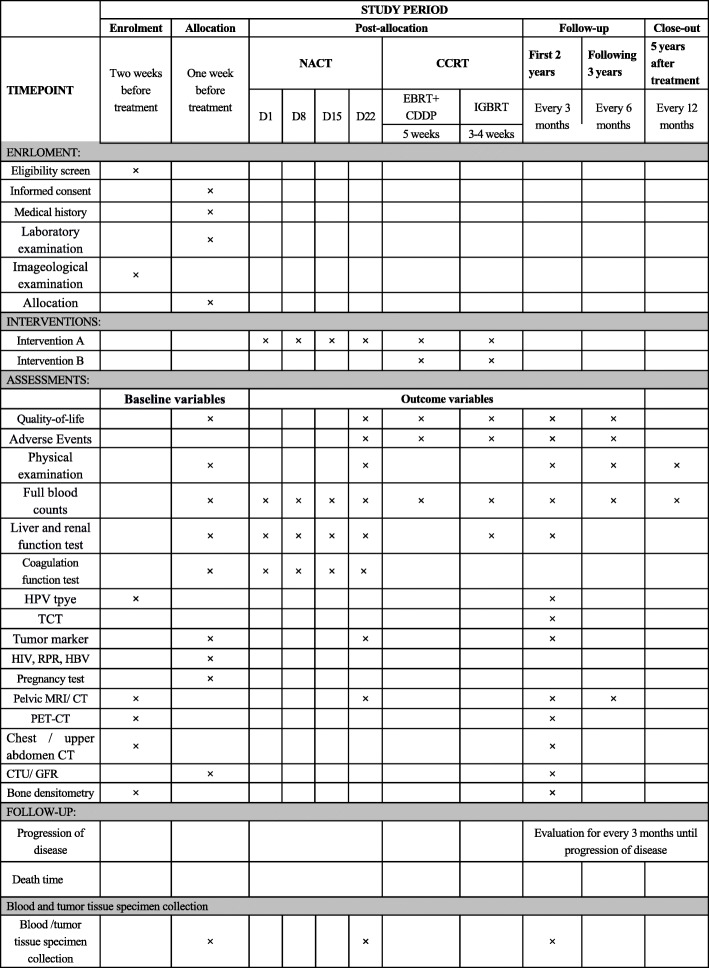
Study period

Participants will be randomly assigned to the study arm or control arm in a 1:1 ratio (Figs. 1 and 2). The randomization sequence will be generated in the SAS software (V.9.4) (SAS Institute Inc., USA) using blocked randomization, stratified by site. The information of randomization was put into opaque sealed envelopes and distributed to each center. The results were entered by a study manager not involved with patient recruitment. During treatment, patients will have gynecological oncologist visits before each day of chemotherapy during NACT and weekly visits during CCRT. Any other anti-tumor medicines, including anti-angiogenesis drugs and immunotherapeutic drugs, are prohibited during this trial unless the recurrence occurs.

#### Clinical data

The data collected will include general medical information (age, childbirth, family history of cancer, and chronic diseases), tumor stage, tumor type, and chemo-/radiotherapy-related adverse events. At baseline, all patients will be physically examined by two gynecological oncologists and will have computed tomography (CT scans) of the chest and upper abdomen or 18-fluoro-2-deoxy-d-glucose positron emission tomography ([18F] FDG-PET) with computed tomography (CT) (PET/CT) and magnetic resonance imaging (MRI)/computed tomography of the pelvis (within the previous 4 weeks). Full blood counts and liver and renal function tests will also be performed, and human papillomavirus (HPV) type and tumor marker (SCCA, CA 125, CA 199) values will be determined. In all cases, a detailed drawing of the lesion will be registered, and glomerular filtration rate (GFR), computed tomography urography (CTU), cystoscopy, and sigmoidoscopy will be performed when necessary. All histology slides will be reviewed by the same panel of pathologists.

#### Control arm

Patients will undergo radiation with concomitant cisplatin (CCRT) treatment as follows: cisplatin (40 mg/m^2^) will be given with hydration over 1 h (before radiation), weekly for 5 cycles. Radiotherapy to the whole pelvis will be given to a total dose of 45 Gy in 5 weeks. Extended fields will be used to treat positive common iliac and/or para-aortic lymph nodes with a total dose of 55–60 Gy. Brachytherapy will be given following the completion of external beam radiation therapy. Patients will receive image-guided adaptive brachytherapy (IGBRT) with a high-risk clinical target volume (HRCTV) of 85 Gy in 3–4 fractions. Patients enrolled were treated by the appointed radiotherapy specialist to ensure quality especially with intensity-modulated radiation therapy (IMRT) and also for IGBRT.

#### Study arm

Neoadjuvant chemotherapy will be given weekly for 4 weeks (on days 1, 8, 15, and 22) as follows: paclitaxel (60 mg/m^2^) over 1 h followed by cisplatin (40 mg/m^2^) over 30 min, given intravenously with hydration for 3 days (3000 ml per day, from the day before the dose to the day after the dose). Both drugs will be delayed if neutrophils are < 1.5 × 10^9^/L and/or platelets are < 75 × 10^9^/L on the day of treatment until hematologic improvement to grade 1 is achieved. The doses of paclitaxel and cisplatin will be reduced by 50% if neutrophils are 0.5–1.0 × 10^9^/L and/or platelets are 25–50 × 10^9^/L. In the event of further hematological toxicity (neutrophils < 0.5 × 10^9^/L and/or platelets < 25 × 10^9^/L), NACT will be discontinued. Patients with a significant hypersensitivity reaction to paclitaxel or cisplatin will be withdrawn from the study. If NACT is discontinued early, patients who proceed to CCRT will commence when blood counts have recovered. Within 2 weeks after NACT, patients will be treated subsequently by CCRT as the control arm as soon as hematological recovery permits.

#### Follow-up

After completion of the treatment, patients will be followed up every 3 months during the first 2 years and every 6 months for the next 3 years. Gynecological oncologist examination will be repeated after NACT, 12 weeks after CCRT and again 3–6 months after treatment. Abdominal ultrasound or upper abdomen CT, pelvic MRI, and tumor marker values will be evaluated. PET/CT, GFR, and CTU will be performed when necessary.

### Study outcomes

#### Primary endpoint

The primary aim of this trial will be to compare the 2-year disease-free survival (DFS) difference between dose-dense NACT followed by CCRT and CCRT alone. We will test the hypothesis: compared with CCRT alone, dose-dense NACT followed by CCRT can significantly improve 2-year DFS in stage IIB–IVA cervical cancer patients.

#### Secondary endpoint

Secondary outcomes include the following:

1. Five-year overall survival (OS) and disease-free survival (DFS) rates. We will test the hypothesis: compared with CCRT alone, dose-dense NACT followed by CCRT can significantly improve 5-year OS and DFS in stage IIB–IVA cervical cancer patients.
2. The response rate 3 months after treatment completion. We will test the hypothesis: dose-dense NACT followed by CCRT will have a higher response rate at 3 months than CCRT alone.
3. The response rate for NACT.
4. Also, we will evaluate the outcome by various stratification factors such as initial tumor volume, nodal status, and response to NACT.
5. Adverse effects and quality of life between the two groups. All grade III/IV acute and late toxicities will be considered significant. We will measure the quality of life with the EORTC QLQ-30 (V3.0) and CX24. We will test the hypothesis: there will be no difference in the incidence and severity of therapy-attributed adverse events between the two groups. Dose-dense NACT followed by CCRT will result in a higher quality of life than CCRT alone.
6. Other outcomes include identifying the predictive biomarkers of treatment response. By collecting tissue and blood samples, including samples from recurrent tumors, before and after NACT and before and after CCRT (if there is residual tumor available after treatment), we intend to compare the differences in the expression of protein, mRNAs, and immune microenvironment in responding and nonresponding subgroups in each treatment arm. We will test the hypothesis: there will be differences in the mRNAs, protein expression, and immune microenvironment in responding and nonresponding groups, independent of the treatment group assignment.

#### Clinical response

Clinical remission will be evaluated according to the World Health Organization (WHO) criteria, the Response Evaluation Criteria in Solid Tumors (RECIST) [14]. A pelvic MRI will be performed at the end of the 4 weeks of NACT to assess the response using the RECIST 1.1 criteria. The overall response will be determined using pelvic MRI 12 weeks after the completion of CCRT. MRI scans will all be reviewed at Ruijin Hospital and assessed by two radiologists blinded to the treatment option based on the RECIST 1.1 criteria. Further radiological assessments will be conducted as clinically indicated. The complete response (CR) is defined as the disappearance of all target lesions, and any pathological lymph nodes (whether target or nontarget) must have a reduction in the short axis to < 10 mm. Partial remission (PR) is defined as at least a 30% decrease in the sum of the diameters of the target lesions, taking as the reference of the baseline sum of the diameters. Progressive disease (PD) is defined as at least a 20% increase in the sum of the diameters of the target lesions, taking as the reference the of baseline sum of the diameters, with the smallest sum of increase at least 5 mm. In addition, a relative increase of more or new lesions is also considered progression. Stable disease (SD) is defined as neither sufficient shrinkage to qualify for PR nor sufficient increase to qualify for PD. Patients with CR or PR will be classified as clinical responders, and patients with stable disease and progression of disease will be defined as clinical nonresponders. If the two radiologists reached different conclusions, the results will be rechecked and discussed to reach a consensus.

#### Adverse effects

The adverse effects of adjuvant chemotherapy and chemoradiation will be evaluated using the Common Terminology Criteria for Adverse Events (CTCAE) version 4.0. All patients will be monitored for marrow function suppression as well as gastrointestinal and dermatologic side effects. Full blood counts will be performed weekly during NACT and twice weekly during CCRT. Biochemistry and toxicity assessments will be carried out weekly during treatment, then 3 months post-CCRT and 12 months for 5 years. A bone density test will be performed before treatment and 3 months after CCRT. Adverse events will be based on the maximum toxicity grade for each type of event. All grade 3/4 acute and late toxicities will be considered significant. Serious adverse events need to be addressed urgently and should be reported to the functionary within 24 h. Adverse events occurring 3 months after the end of treatment will be considered late toxicity. Quality of life will be assessed with the EORTC QLQ-30 (V3.0) and CX24 before treatment, post-NACT, post-CCRT, 3 months post-CCRT, and every 12 months for 5 years.

### Statistical analysis

Baseline characteristics (age, tumor size, tumor stage, etc.) for both treatment groups will be compared to ensure comparability of the patient populations to rule out selection bias. The means and standard deviations will be used to report continuous variables, and proportions will be used to report categorical variables. Comparisons of categorical outcome measures such as response rate, survival rate, and incidence of adverse effects will be computed using the chi-square test or Fisher’s exact test if any of the expected values in the contingency table are less than 5. Kaplan-Meier survival analysis will be used for 2-year and 5-year overall survival and disease-free survival comparisons. Relative risks, or odds ratios, with their corresponding 95% confidence intervals will be analyzed by multivariable logistic regression. A *P*-value of < 0.05 was considered significant.

## Discussion

Despite the availability of an effective screening program and the papillomavirus vaccine, a large proportion of patients are not discovered to have cervical cancer until they have reached an advanced stage and suffer from local recurrences and distant metastases. The Cochrane meta-analysis reported a stage-dependent advantage of CCRT over radiotherapy (RT), with 5-year survival benefits of 10% for women with stage IB to IIA cervical cancer, 7% for women with stage IIB cervical cancer, and 3% for women with stage III to IVA cancer [15]. Improving the survival of these patients is an urgent issue.

Some researchers have studied the effect of NACT prior to CCRT. Studies have shown traditional thrice-weekly platinum-based NACT followed by CCRT has been applied to LACC patients. Narayan et al. [9] retrospectively compared the effect of 2 cycles of thrice-weekly TPF (cisplatin + paclitaxel + 5-flurical) or TF (cisplatin + 5-flurical) followed by CCRT vs. CCRT alone in 723 stage IIB–IIIB cervical cancer patients. They found that NACT followed by CCRT could improve 5-year progression-free survival (58.3% vs. 41.8%) but had no impact on the overall survival. Marita et al. [16] retrospectively analyzed the survival of 207 stage IIB–IIIB cervical cancer patients who received 2–4 cycles of platinum-based NACT prior to CCRT. The results revealed that the 5-year survival rates for stage IIB–IIIA and IIIB were 84% and 61%, respectively, which are superior to the survival rates of traditional CCRT reported in the literature. A randomized open-label phase II trial enrolled 107 patients, 55 randomly assigned to the NACT arm and 52 to the CCRT-alone arm. NACT was associated with an inferior 3-year PFS (40.9% vs. 60.4%), a lower 3-year OS rate (60.7% vs. 86.8%), and a lower complete response rate (56.3% vs. 80.3%) [10].

In addition, a meta-analysis of 21 randomized trials showed no increase in OS despite a significant reduction in tumor volume by primary chemotherapy in a comparison of radiotherapy alone with radiotherapy preceded by chemotherapy. However, subgroup analysis showed a 7% improvement in the 5-year OS with a chemotherapy cycle length of < 14 days over that shown in studies with longer cycle lengths, and cisplatin dose intensities ≥ 25 mg/m^2^ per week tended to show a survival advantage [8]. Regarding the distribution and metabolism of these drugs, Koshiba et al. [17] found that paclitaxel was retained in cervical cancer tissues for 6 days after intravenous administration of a 60-mg/m^2^ dose, but it could not be detected after 2 weeks, suggesting that a weekly schedule was most effective for tumor cell death rather than the standard thrice-weekly regimen. Thus, administering NACT at shorter intervals (dose-dense) may result in enhanced cell death and overcome accelerated repopulation. A dose-dense (weekly) schedule is likely to result in improvement in the outcomes.

The initial results from two phase II studies [11, 12] have been reported on patients who received NACT using weekly paclitaxel (60–80 mg/m^2^) and carboplatin (AUC = 2) for 6 weeks followed by CCRT. Following NACT, a response rate of 67.5–70% was achieved, mostly partial responses. Post-CCRT, 85–100% of eligible patients achieved CR. Grade 3–4 hematologic toxicity was observed in approximately 20% of patients. A 3-year overall survival rate of 68% was observed in 42 stage IB2–IVA patients. These observations are encouraging.

The approach is now being evaluated in a randomized trial (CRUK/11/024: INTERLACE trial) (http://www.clinicaltrials.gov). The combination of paclitaxel and carboplatin has been used in a weekly schedule; this combination maximizes the potential additive/synergistic interactions with different mechanisms of action. However, a phase II nonrandomized trial reported that two cycles of weekly cisplatin (35 mg/m^2^) combined with gemcitabine (1000 mg/m^2^) as an upfront treatment for IB2–IVA patients managed with CCRT did not result in a meaningful improvement in ORR [18].

Which NACT regimen is the best? Regarding chemotherapy in the treatment of advanced metastatic or recurrent cervical cancer, cisplatin has been considered the most effective agent. The cisplatin/paclitaxel (TP) combination is superior to other regimens. In a phase III trial comparing four regimens (cisplatin/paclitaxel, cisplatin/vinorelbine, cisplatin/gemcitabine, cisplatin/topotecan), cisplatin/ paclitaxel appears superior to the others [19]. According to the results from JCOG0505, among patients who had not received prior cisplatin, TP resulted in longer overall survival than TC (23.2 months vs. 13.0 months) [13].

Our trial is similar to the ongoing INTERLACE trial in that we both aim to investigate whether additional induction chemotherapy given on a weekly schedule immediately before standard chemoradiation leads to an improvement in the overall survival and disease-free survival, and the protocol for concurrent chemoradiation (CCRT) was alike. However, the chemotherapy regimen and the targeted populations are different. The chemotherapy regimen of the INTERLACE trial was 6 cycles of weekly paclitaxel (80 mg/m^2^) and carboplatin (AUC 2). Based on these data and the differences in our population, we consider weekly paclitaxel (60 mg/m^2^) and cisplatin (40 mg/m^2^) for 4 weeks as the first-line choice for the NACT regimen. In addition, the recruit criteria are slightly different: the INTERLACE trial will recruit FIGO 2009 stage Ib2–IVa (except stage IIIA, stage IB1, and positive lymph nodes are also eligible) cervical carcinoma patients. Our trial will recruit FIGO 2018 stage IIB–IVA patients. The primary endpoint of this trial is to compare the 2-year disease-free survival (DFS) difference between dose-dense NACT followed by CCRT and CCRT alone. However, the INTERLACE trial compared the 5-year overall survival (OS) difference between the two arms as the primary endpoint.

We aim to conduct a randomized trial to compare the 2-year DFS of this NACT regimen followed by CCRT with those of CCRT alone for newly diagnosed stage IIB–IVA cervical cancer patients. The survival outcomes, response rates, adverse effects, and quality of life will also be assessed, and potential biomarkers for predicting treatment response will be studied. If a superior therapeutic effect is confirmed, NACT with weekly TP followed by the CCRT procedure may become an optimized method of cancer treatment.

## Trial status

The protocol version number and date: version 1.0, December 2018 (revised version 1.1, August 2020)

The date recruitment began: 01 January 2019

The approximate date when recruitment will be completed: 31 December 2026

## Data Availability

The datasets used or analyzed during the current study are available from the corresponding authors on reasonable request.

## Abbreviations

CCRT: Concurrent chemoradiation
NACT: Neoadjuvant chemotherapy
IGBRT: Image-guided adaptive brachytherapy
LACC: Locally advanced cervical cancer
OS: Overall survival
PA: Para-aortic
EFR: Extended field radiotherapy
AUC: Area under the curve
ECOG: Eastern Cooperative Oncology Group
WBC: White blood cell
HB: Hemoglobin
ALT: Alanine aminotransferase
AST: Aspartate aminotransferase
ALP: Alkaline phosphatase
Cr: Creatinine
HIV: Human immunodeficiency virus
CT: Computed tomography
FDG-PET: 18-Fluoro-2-deoxy-d-glucose positron emission tomography
PET-CT: 18-Fluoro-2-deoxy-d-glucose positron emission tomography with computed tomography
MRI: Magnetic resonance imaging
HPV: Human papillomavirus
SCCA: Squamous cell carcinoma antigen
CA 125: Carbohydrate antigen 125
GFR: Glomerular filtration rate
CTU: Computed tomography urography
HRCTV: High-risk clinical target volume
DFS: Disease-free survival
WHO: World Health Organization
RECIST: Response Evaluation Criteria in Solid Tumors
CR: Complete response
PR: Partial remission
PD: Progression disease
SD: Stable disease
CTCAE: Common Terminology Criteria for Adverse Events
RT: Radiotherapy
TPF: Cisplatin/paclitaxel/5-flurical
TF: Cisplatin/5-flurical
TP: Cisplatin/paclitaxel
TC: Carboplatin/paclitaxel
